# Determining gene flow and the influence of selection across the equatorial barrier of the East Pacific Rise in the tube-dwelling polychaete *Alvinella pompejana*

**DOI:** 10.1186/1471-2148-10-220

**Published:** 2010-07-22

**Authors:** Sophie Plouviez, Dominique Le Guen, Odile Lecompte, François H Lallier, Didier Jollivet

**Affiliations:** 1 Université Pierre et Marie Curie-Paris 6, Laboratoire Adaptation et Diversité en Milieu Marin, Roscoff, France; 2 CNRS, UMR 7144 Station Biologique de Roscoff, BP 74, Place Georges Teissier, 29682, Roscoff, France; 3 Duke University Marine Laboratory, 135 Marine Lab Road, 28516, Beaufort NC, USA; 4 IGBMC, Laboratoire de Bioinformatique et de Génomique Intégratives, Parc de l'Innovation, Campus Illkirch, Strasbourg, France

## Abstract

**Background:**

Comparative phylogeography recently performed on the mitochondrial *cytochrome oxidase I *(*mtCOI*) gene from seven deep-sea vent species suggested that the East Pacific Rise fauna has undergone a vicariant event with the emergence of a north/south physical barrier at the Equator 1-2 Mya. Within this specialised fauna, the tube-dwelling polychaete *Alvinella pompejana *showed reciprocal monophyly at *mtCOI *on each side of the Equator (9°50'N/7°25'S), suggesting potential, ongoing allopatric speciation. However, the development of a barrier to gene flow is a long and complex process. Secondary contact between previously isolated populations can occur when physical isolation has not persisted long enough to result in reproductive isolation between genetically divergent lineages, potentially leading to hybridisation and subsequent allelic introgression. The present study evaluates the strength of the equatorial barrier to gene flow and tests for potential secondary contact zones between *A. pompejana *populations by comparing the *mtCOI *gene with nuclear genes.

**Results:**

Allozyme frequencies and the analysis of nucleotide polymorphisms at three nuclear loci confirmed the north/south genetic differentiation of *Alvinella pompejana *populations along the East Pacific Rise. Migration was oriented north-to-south with a moderate allelic introgression between the two geographic groups over a narrow geographic range just south of the barrier. Multilocus analysis also indicated that southern populations have undergone demographic expansion as previously suggested by a multispecies approach. A strong shift in allozyme frequencies together with a high level of divergence between alleles and a low number of 'hybrid' individuals were observed between the northern and southern groups using the *phosphoglucomutase *gene. In contrast, the *S-adenosylhomocysteine hydrolase *gene exhibited reduced diversity and a lack of population differentiation possibly due to a selective sweep or hitch-hiking.

**Conclusions:**

The equatorial barrier leading to the separation of East Pacific Rise vent fauna into two distinct geographic groups is still permeable to migration, with a probable north-to-south migration route for *A. pompejana*. This separation also coincides with demographic expansion in the southern East Pacific Rise. Our results suggest that allopatry resulting from ridge offsetting is a common mechanism of speciation for deep-sea hydrothermal vent organisms.

## Background

Many population genetic models have been proposed to describe how individuals disperse between geographically separated localities including n-island models [1,2], stepping-stone [3] or isolation-by-distance [4,5] models, and have subsequently been adapted to a metapopulation context to integrate population dynamics through time (i.e. local extinction and recolonisation rates; reviewed in [6,7]). Nevertheless, a physical barrier to dispersal can disrupt the relationship between genetic differentiation and migration rate: all models show that the establishment of a long-term physical barrier to dispersal abruptly reduces or stops gene flow between populations and leads to a rapid change in allele frequencies on either side of the barrier that may be dampened or accelerated depending on the type of selection at specific loci. This often leads to the fixation of mutations (divergence) and reciprocal monophyly for many loci, provided that no lineage extinction or population admixture occurs [8]. Secondary contact events can produce genetic incompatibilities and clinal allelic distributions whose shapes depend primarily on the strength of selection against hybrids [9].

Deep-sea hydrothermal vents represent a patchily distributed habitat (sometimes separated by thousands of kilometres) suggestive of a one-dimensional stepping-stone model over 60 000 kilometres of globe-encircling ridge crests [10]. Because of their dependence on 'hot' sulphidic fluids from vent chimneys, deep-sea hydrothermal vent species display an island-like distribution along ridges. This habitat distribution raises questions about vent species' dispersal among sites and the putative occurrence of physical barriers to dispersal [11,12]. It has been hypothesised that hydrothermal vent species disperse primarily in their local neighbourhood [13], as in the Kimura and Weiss [3] one-dimensional stepping-stone model, and thus follow an isolation-by-distance model [11]. However, in most cases, allozyme studies have failed to detect isolation-by-distance among populations of hydrothermal vent species (e.g. [14,15]), suggesting that an n-island model [1] is a better description of gene flow in vent systems.

Amongst vent species, the deep-sea hydrothermal vent polychaete *Alvinella pompejana*, which only lives at the top of hydrothermal-vent chimneys of the East Pacific Rise (EPR) between 38°S and 27°N (Guaymas Basin), has been described as a pioneer species because it is the first species to colonise new hot-sulphide edifices [16]. The larval development of this tube-dwelling worm is still largely unknown, but large yolk-rich eggs (150-200 μm), suggest that larval development is lecithotrophic and could be delayed in cold (1.5-2°C) water away from vent fields [12]. This worm species reproduces via internal fertilization and has nearly continuous gametogenesis, maximising its reproductive effort [17]. During mating, spermatozoids are transferred to the female in a spermatecae, and multiple paternity is possible [18]. This kind of reproductive mode is known to strongly favour larval retention in the immediate surrounding of the source population, which may be advantageous in unstable and patchy environments [19]. Given this set of reproductive characteristics and the recurrent extinction and recolonisation of vent edifices, Jollivet *et al. *[20,21] have proposed that this species follows a propagule-flux model of dispersal (i.e. (re)colonisation of a previously extinct or new vent site from the closest populations, [22]). A hydrodynamic model predicts that effective migration rates - accounting for *A. pompejana*'s larval biology - cannot be maintained across vent fields if larvae travel more than 15-30 days in the water column [12]. Nevertheless, using allozymes this species lacks any clear genetic structure from latitudes 9°50'N to 21°N despite a microgeographical population differentiation at the scale of vent fields (i.e. tens of kilometres) [23]. These results suggest that the potential for larvae to disperse may be higher than previously thought provided that no physical barrier to gene flow exists along the ridge system [24]. Accordingly, Pradillon *et al. *[25] reported an experiment where *A. pompejana *embryos can arrest their development in cold water, potentially delaying their metamorphosis during dispersal from vents. To better explain the absence of isolation-by-distance in the northern EPR, three hypotheses have been proposed: (1) allozymes lack the power to detect genetic differentiation between populations; (2) balancing selection - due to the homogeneity of vent conditions along the whole ridge system - maintains homogeneous allozyme frequencies; or (3) displacement of habitat patches over geological times promote metapopulation dynamics, thereby maintaining gene flow [24]. The first hypothesis was tested by Audzijonyte and Vrijenhoek [26] who suggested that undersampling biased some of the previous analyses. The second hypothesis was supported by Piccino *et al. *[27] who observed balancing selection on the phosphoglucomutase (PGM) locus with one particularly thermostable allele being more frequent in young populations. Interestingly, this study also showed allele frequencies were homogenous at the metapopulation scale, proving support for the third hypothesis. The third hypothesis was tested by a model which showed that bursts of colonisation events following the emergence of new vent sites along with population reconnections were sufficient to prevent genetic differentiation between distant sites given limited dispersal of *A. pompejana *larvae [21]. The model predictions were however, based on the possibility that new vent sites could occur across physical barriers such as transform faults.

Recently, genetic divergence of nearly 1% was observed at the mitochondrial *cytochrome c oxidase subunit I *(*mtCOI*) gene between the northern and southern EPR populations of *A. pompejana *[28,29], suggesting the occurrence of an impermeable equatorial barrier to gene flow. Furthermore, Plouviez *et al. *[29] demonstrated that this barrier affected most EPR vent fauna 1 to 2 Mya, giving credence to the role of transform faults in provoking vicariant events between vent communities and allopatric speciation. In the present study, an extended analysis of allozyme and *mtCOI *haplotype distributions over the geographic range of the species, including the southern EPR, was performed to better address the ability of allozymes to detect genetic differentiation and to discriminate between the three hypotheses described above. Three additional nuclear genes, including the *PGM *locus, were used to evaluate the strength of this equatorial barrier to gene flow and to test for potential secondary contact. We also attempted to quantify migration and/or introgression rates between the previously isolated northern and southern EPR populations to disentangle the effects of selective and demographic processes contributing to the genetic break.

## Methods

### Collection

**Figure 1:**
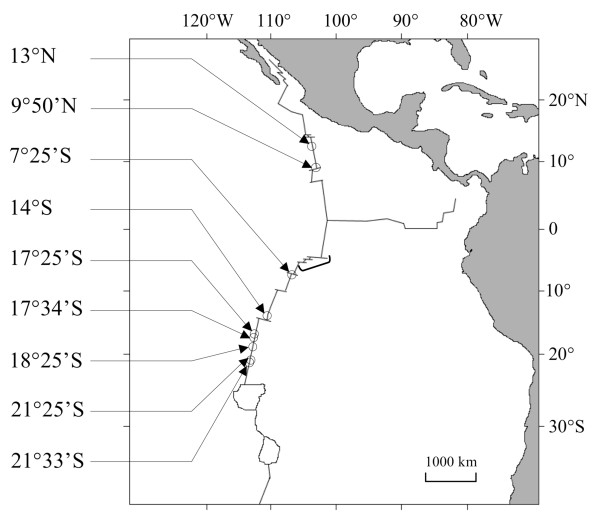
**Map of the south-east Pacific and *Alvinella pompejana *sampling locations along the East Pacific Rise**. Dots represent sampled hydrothermal vent fields. The bracket between 9°50'N and 7°25'S indicates the Quebrada, Discovery and Gofar transform faults from north to south, respectively.

*Alvinella pompejana *polychaetes were collected with the manned submersible *Nautile *during two oceanographic cruises in 1999 (HOPE) and 2002 (PHARE) at northern EPR sites (Figure 1) and one cruise in 2004 (BIOSPEEDO) at southern EPR sites from latitudes 7°25'S to 21°33'S. Onboard (R/V *L'Atalante*), samples were measured at the fourth setigerous segment, sexed, and cut in two: the cephalic portion was frozen immediately at - 80°C for allozyme analyses and the body was preserved in 95% ethanol for DNA analyses.

### Allozyme genotyping

Allozymes, encoded by nine enzyme systems, were examined from seven hydrothermal vent field samples (Table 1), following the protocols of Pasteur *et al. *[30]. Proteins from each frozen gill tissue were extracted according to the method in Piccino *et al. *[27]. Allozyme electrophoreses were conducted on 12% starch gels using three different buffer systems: (1) Tris-citrate pH 8.0 (TC 8.0) for phosphoglucomutase (PGM, E.C. 5.4.2.2), mannose phosphate isomerase (MPI, E.C. 5.3.1.8), 6-phosphogluconate dehydrogenase (6PGD, E.C. 1.1.1.44) and glucose phosphate isomerase (GPI, E.C. 5.3.1.9); (2) Tris-HCl pH 8.5/8.2 (THCl 8.5) for triose phosphate isomerase (TPI, E.C. 5.3.1.1), leucine amino peptidase (LAP, 3.4.11.1) and hexokinase-1 (HK, 2.7.1.1); and (3) Tris-citrate pH 6.7/6.3 (TC 6.7) for acid phosphatase (ACP, E.C. 3.1.3.2) and isocitrate dehydrogenase (IDH, E.C. 1.1.1.42).

**Table 1:** Location of individuals and sample size for allozymes (n_allo_), *mtCOI *(n_*mtCOI*_), and nuclear genes.

Vent field	Geographic position	Depth (m)	n_allo_	n_*mtCO*I_	n_*GlobX*_	n_*SAHH*_	n_*PGM*_	n_RFLP_
13°N	12°43-50'N	2560-2700	40	67	19	17	20	74
	103°53-57'W				**10**	**11**	**12**	
9°50'N	9°31-51'N	2500-2585	0	10	7	20	8	0
	104°15-18'W				**4**	**15**	**6**	
7°25'S	7°25'S	2735-2752	17	14	14	13	15	12
	107°47-49W				**9**	**8**	**11**	
14°S	13°59'S	2623-2632	15	20	16	16	15	16
	112°29'W				**9**	**14**	**11**	
17°25'S	17°25'S	2578-2590	34	30	15	16	10	15
	113°12'W				**10**	**9**	**8**	
17°34'S	17°35-36'S	2591-2597	19	36	18	15	12	17
	113°15'W				**12**	**10**	**10**	
18°25'S	18°26-37'S	2636-2680	52	29	17	17	14	14
	113°23-24'W				**11**	**14**	**10**	
21°25'S	21°25'S	2804-2831	12	30	16	16	13	22
	114°16-17'W				**12**	**11**	**10**	
21°33'S	21°33'S	2771-2846	23	25	14	20	17	11
	114°16-18'W				**10**	**14**	**13**	

n_allo_: number of individuals genotyped for allozymes, n*_mtCOI_*: number of individuals sequenced for the *mtCOI *gene, n*_GlobX_*, n*_SAHH_*, n*_PGM_*: number of recaptured sequences for each nuclear gene using a maximum of two consensus sequences (alleles) per individual with the number of recaptured individuals given in bold, n_RFLP_: number of genotyped individuals for *PGM *RFLPs.

### DNA sequencing

Genomic DNA was extracted using a CTAB extraction procedure following the protocol described in Plouviez *et al. *[29]. *A. pompejana mtCOI *sequences from this previous study and the sequences of the outgroup *A. caudata *were obtained with the 'universal' LCOI and HCOI primers [31] using the previously described sequencing protocol. Specific primers (additional file 1) were designed to amplify partial exon-intron sequences of three nuclear genes, either identified during transcriptome sequencing (the *S-adenosylhomocysteine hydrolase *(*SAHH*) and *globin X *(*GlobX*) genes [32]), or from the complete *phosphoglucomutase *gene sequence in *A. pompejana *previously obtained using degenerate primers (D.J., unpublished data). A non-synonymous change was discovered in exon 4 of *PGM *and specifically targeted in subsequent analyses. Sizes of the DNA fragments and their exon-intron structures (boundaries) are given in additional file 2.

Polymerase chain reactions (PCR) and cloning were performed using the mark-recapture (MR) method developed by Bierne *et al. *[33] with 4-nucleotide tails added to the 5' end of each primer. Each individual was amplified by PCR separately with a different combination of 5'-tailed primers. PCR were conducted in a 25 μl volume that included 1X buffer (supplied by manufacturer), 2 mM MgCl_2_, 0.05 mM (0.25 mM for *PGM*) of each dNTP, 0.48 μM (0.4 μM for *PGM*) of each primer (additional file 1), 0.5 U of Taq polymerase (Thermoprime plus), 5 μl of template DNA and sterile H_2_O. Thermal cycling parameters used an initial denaturation step at 94°C for 3 min, followed by 40 cycles at 94°C for 30 s, T_a _for 20 s and 72°C for 2 min 30 s, before a final 10 min extension at 72°C. As per the MR protocol, eight PCR-products were pooled together per cloning experiment with two cloning experiments per vent locality (16 individuals per sampled site). PCR products were isostochiometrically mixed, purified using QIAQuick™ columns, and ligated into a pGEM-T vector (pGEM-T cloning kit, Promega, Madison, WI, USA). For each cloning experiment, 32 positive clones were sequenced on both strands at Genoscope (Evry) with universal plasmid primers (SP6 and T7), leading to a total number of approximately 3500 nuclear sequences. Sequences from all genes were proofread using CodonCode Aligner 2.0.6 http://www.codoncode.com/aligner/. Sequence alignments were initially performed with ClustalW [34] in BioEdit version 6.0.6 [35] and adjusted manually. For nuclear genes, *in vitro *recombinants between individuals from the same cloning set were detected by their abnormal combination of 5'-tails and removed from the dataset. Multiple recaptures also allowed us to discard intra-individual *in vitro *recombinants (PCR recombination of heterozygous alleles) and artefactual/somatic mutations.

### Detecting a putative barrier to gene flow

Linkage disequilibrium between allozyme loci was examined for each locality and for the complete dataset using Genetix version 4.05.2 [36] to detect redundant information. For each locality, allele frequencies were then calculated for each locus and departures from Hardy-Weinberg equilibrium were estimated using the Weir and Cockerham [37]'s estimator *f *and tested with 1000 permutations. To detect putative barriers to gene flow, genetic differentiation was estimated along the EPR from a multilocus Fst as calculated using Weir and Cockerham's *θ *[37], adding populations one at a time from the most southern locality (21°33'S) to 13°N. At each step, the F index was tested with 1000 permutations of the dataset. Exact G test was performed using Genepop version 4.0.10 [38] for all populations.

Isolation-by-distance was tested along the whole EPR using a Mantel Spearman test [39] with 5000 permutations using Genepop version 4.0.10 [38]. Because previous studies [28,29] detected a barrier to gene flow across the Equator for the *mtCOI *marker, this test was also performed without the 13°N population. To better understand population structure, the number of genetically-differentiated groups (*K*) was evaluated using individual assignments to these *a priori *groups based on their multilocus allozyme genotypes from an admixture model with a 150 000 burn-in length period followed by 50 000 MCMC repetitions. The most appropriate number was obtained by testing *K *values from 1 to 15 using the Structure program (version 2.2) [40]. Convergence of the Markov chain simulations was checked for stationarity by monitoring ten iterations.

Average recapture rates were 0.60, 0.74 and 0.63 for *GlobX*, *SAHH *and *PGM *genes, respectively. For all statistical analyses based on recaptured sequences, one consensus allele was randomly selected per individual to avoid any putative bias due to the cloning method. Allele diversity *Hd*, nucleotide diversity *π_N_*, and Watterson's theta *θ_W _*were estimated for northern and southern EPR sites separately with DNAsp 4.10.3 [41]. For each gene, allele networks were constructed using Network 4.5.1.0 [42] to search for divergence across the Equator (all recaptured alleles were used). Genetic differentiation indexes (ø*_st_*) were computed using Arlequin 3.1 [43] from nucleotide sites by grouping populations from either side of the Equator and departures from zero (no differentiation) were tested using 1000 permutations.

Because most coalescent-based approaches impose strong assumptions on the absence of recombination, recombinants were detected by the Hudson and Kaplan [44]'s four-gamete test, using DNAsp 4.10.3, and discarded from the dataset for the IMa and Sweep_bott analyses.

### Detection of gene flow between the two geographic groups of *A. pompejana *using an isolation-with-migration model

Population sizes, migration rates, and the time of population splitting were estimated using the isolation-with-migration model (IMa software, [45]) by grouping populations from each part of the geographical barrier (i.e. 9°50'N and 13°N on one side and the remaining southern populations on the other side). This model allowed us to calculate posterior density probabilities of these three parameters assuming that migration could still occur after the splitting event of an ancestral population into two descendant populations. To check convergence of the Markov chain Monte Carlo (MCMC) with the true stationary distribution, multiple runs were performed using different starting points and autocorrelation between parameter values was assessed over the course of the runs. Upper bounds of the uniform prior distributions used for the final run were: q = 30 (prior population size parameters), m = 1 (migration rates) and t = 2 (divergence time). A swapping procedure was also used to enhance the mixing of chains using a geometric heating scheme with 15 parallel chains. Inheritance scalars were assigned to 1 for nuclear genes (autosomal) and 0.25 for *mtCOI *to adjust for their expected population sizes. The Hasegawa-Kishino-Yano model [46] was chosen to allow for multiple substitutions at the same site.

To better estimate the date since the splitting event, IMa parameters of divergence between populations (t_IMa_) were converted into years. Because mutation rates have not yet been estimated for alvinellid polychaetes, with the exception of the *mtCOI *gene [47] which is known to have a higher mutation rate compared to nuclear genes, a geometric mean was derived from *mtCOI*, as well as *PGM *and *SAHH *for which a mutation rate has been obtained from the hydrothermal vent gastropod *Lepetodrilus fucensis *across the Blanco transform fault [48] and the hydrothermal vent mussels *Bathymodiolus thermophilus *across the Easter microplate [49], respectively. The *GlobX *gene was not used because there is no adequate calibration for its mutation rate. Before calculating the geometric mean, an average mutation rate was assessed per locus per generation accounting for different lengths of the three genes (*mtCOI *507 bp, *PGM *940 bp, *SAHH *531 bp), assuming one generation per year. The geometric mean U = 0.64 × 10^-6^, close to the value previously found using eight genes (*mtCOI *and 7 nuclear genes, U = 0.63 × 10^-6^) on *B. thermophilus *[49], was then used to recalibrate the divergence time from IMa to time in years (T = t_IMa _/U).

### Detecting putative 'hybrids' between the north and south EPR using the PGM gene

Because substantial divergence has been observed between the northern and southern EPR clades at the nuclear *PGM *gene, fixed mutations were used to detect and locate 'hybrids' between these two geographic regions (heterozygous individuals with one allele from the northern clade and one allele from the southern clade or individuals sampled in a geographic region with alleles typical of the clade of the other region). A restriction fragment length polymorphism (RFLP) analysis combined with genotyping insertion-deletions (indels) was performed to create a composite *PGM *genotype for each individual. Among the four clades observed, clade 3 displayed two restriction sites for the *Rsa*I enzyme whereas the three remaining clades only had one. Clade 4 displayed two restrictions sites for *Ssp*I whereas the three remaining clades have three sites. Clades 1 and 2 differed by several fixed substitutions for which no restriction site was observed and the presence of a 9 bp indel. *Rsa*I and *Ssp*I digestions were performed on *PGM *PCR products following manufacturer's protocols and the presence/absence of the indel was genotyped using new nested primers defined from each side of the indel. Individuals were amplified in 15 μl solutions that included 1X buffer (supplied by manufacturer), 1.25 mM MgCl_2_, 0.2 mM each dNTP, 0.1 μM of fluorescent-labelled (IRD700™) forward primer, 0.27 μM of unlabelled forward primer, 0.33 μM of unlabelled reverse primer (additional file 1), 0.5 U of Taq polymerase (Thermoprime plus), 4 μl of template DNA and sterile H_2_O. Thermal cycling parameters used an initial denaturation at 94°C for 2 min, followed by 30 cycles at 94°C for 30 s, annealing temperature (T_a_, see additional file 1) for 30 s and 72°C for 25 s, before a final 10 min extension at 72°C. PCR products were then electrophoresed on a denaturing acrylamide 41 cm gel in a Li-Cor NEN Global IR2 DNA analyzer.

### Detecting demographic and/or selective events from coalescence trees

Because gene genealogies contain information on demographic and/or selective events, neighbour-joining trees (distances obtained by the maximum composite likelihood) were performed using Mega 4.0.2 [50], separately on northern and southern EPR populations and rooted with an *A. caudata *sequence for non-ambiguous portions of each gene. Departures from neutral expectations were assessed by estimating Tajima (*D*, [51]), Fu and Li (F*, [52]) and Fay and Wu (*H*, [53]) indexes in each group of populations to test for putative demographic and/or selective events. A multi-locus HKA test [54] was computed for each group with one sequence of *A. caudata *used as an outgroup using the HKA software (J. Hey's web page: http://lifesci.rutgers.edu/~heylab/HeylabSoftware.htm#HKA). This test compares divergence and polymorphism at several loci to determine if any loci display a departure from neutral evolution. McDonald Kreitman tests (MK, [55]) were also performed to test for selection. We tested for the fit of either a bottleneck or a selective sweep model for all four loci (including *mtCOI*) in northern and southern EPR populations (excluding introgressed alleles) using Sweep_bott [56] with 100 000 first-step iterations, 1 000 000 second-step iterations and 20 optimisation processes with a theta range from 1 to 30. This test compares three distinct coalescent models (including a null constant-size model: M1) using likelihood ratio tests (LRTs) and estimates whether the strength and the time since bottleneck are sufficiently similar between loci to suggest a historical bottleneck (M2 model). If there is a departure from the M1 model but no detected bottleneck, then the selective sweep model is considered as more probable. A strong selective sweep at a single locus can significantly reduce the fit to a bottleneck model resulting in false rejection of a bottleneck hypothesis. To better discriminate the bottleneck and the sweep models, departure from the M1 model was tested individually for each locus (called M3 model). If all loci showed a departure from M1 model, then a bottleneck would occur even if the sweep model was found more probable using the multilocus approach. If only a few loci gave a departure from this model, then the bottleneck was ruled out and the selective sweep model selected.

## Results

### Equatorial barrier to gene flow

**Figure 2:**
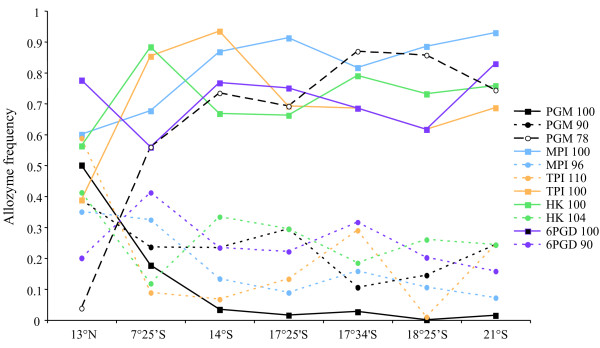
**Allozyme frequency clines along the East Pacific Rise**. Frequencies of the most frequent alleles found in *A. pompejana *populations for PGM, MPI, TPI, HK and 6PGD allozyme loci are plotted according to latitude. Each colour represents a distinct allozyme locus. Individuals from 9°50'N were not used in the analysis simply because they have been only preserved in alcohol.

Linkage disequilibria were not significant for any pair of loci in any population (p > 0.05 using the Garnier-Géré and Dillman [57] test), indicating that each locus behaves independently. The number of alleles ranged from 3 (GPI, IDH, ACP, LAP, HK) to 5 (TPI, see: additional file 3), however most loci displayed only one to 3 alleles at moderate frequency (> 5% in the total population) and were shared among all populations. In contrast, rare alleles were often private (additional file 3). Common alleles reversed in frequency at the TPI locus across the Equator, with the 13°N site showing opposite trends in allele frequencies compared to the 7°25'S site (Figure 2). A similar result was observed at the PGM locus but with a nearly complete replacement of allele 100 by allele 78 and the frequency of allele 90 slightly decreasing from north to south. Significant heterozygote deficiencies (Fis > 0, see additional file 3) were not linked to any particular locus and were not systematically observed at any location. However, a more detailed examination of Fis values at loci with two or three common alleles indicated that the highest values were found at 14°S and 17°25'S.

The multilocus estimate of Weir and Cockerham's *θ *[37] over all populations was not significantly different from zero (*θ *= 0.097, p > 0.05, 1000 permutations) but Fst significantly differed from zero with the Robertson and Hill's correction ([58], *RH *= 0.060, p < 0.05, 1000 permutations). The exact G test for genic differentiation was significant for the overall populations (p < 0.05). Tests for isolation-by-distance showed a significant correlation between the genetic distance of allozymes *θ/(1-θ) *and geographic distance across the entire EPR region (Mantel Spearman test: p = 0.014) but was not significant when the 13°N site was removed from the dataset (p = 0.115). Partitioning genetic structure using the Bayesian clustering method of Pritchard *et al. *[40] identified three genetically distinct groups present in different proportions within each vent field. The first group was primarily made up of individuals from 13°N, whereas the second group clustered individuals mostly from 7°25'S-14°S, and the third group clustered 17°25'S to 21°33'S individuals.

**Figure 3:**
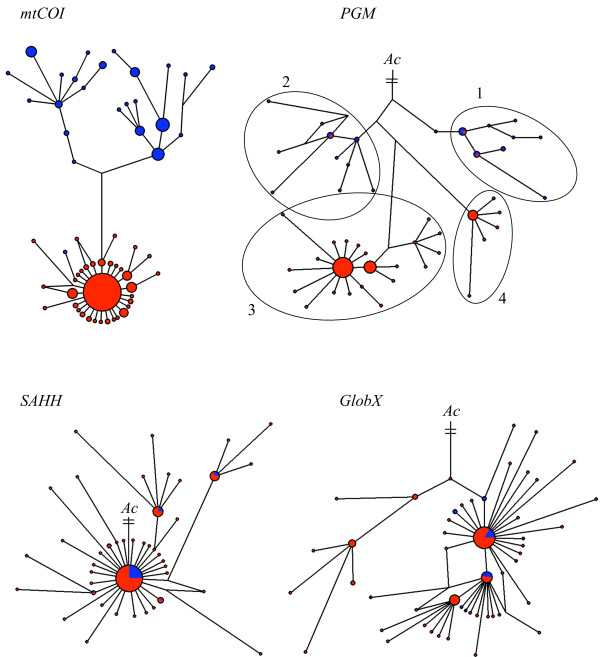
**Median-joining networks of the *mtCOI *and nuclear genes**. Blue and red labels represent individuals sampled from the northern (9°50'N and 13°N) and southern (7°25'S-21°33'S) EPR, respectively. Sizes of haplotype circles and lengths of connecting lines are proportional to the number of sequences studied and the number of mutations that separate two linked haplotypes, respectively. Nuclear genes are rooted by a consensus outgroup sequence of *A. caudata *(connecting line between *A. pompejana *and the *A. caudata *outgroup is not representative of sequence differences due to the high number of mutations).

DNA sequence variation among the three nuclear genes (*SAHH*, *GlobX *and *PGM*) and *mtCOI *were analysed in the northern and southern populations of *A. pompejana *across the Equator (Table 2, Genbank accession numbers: HM183084-HM183493). As shown by Plouviez *et al. *[29], the network of *mtCOI *haplotypes displayed two reciprocally monophyletic clades (~1% divergence) in the northern and southern EPR, respectively (Figure 3). Similarly, the *PGM *locus possessed two sets of two divergent clades that were geographically structured (average northern/southern EPR divergence = 0.76%): clades 1 and 2 were mainly found in the northern EPR, whereas clades 3 and 4 were distributed in the southern EPR (Figure 3). Populations from the northern and southern EPR were significantly different from each other at this locus (ø*_st _*= 0.18, p < 0.01). Although populations appeared to be genetically differentiated across the Equator at the *GlobX *locus (ø*_st _*= 0.04, p < 0.05), these populations were genetically homogeneous across regions at the *SAHH *locus (ø*_st _*value = 0.00, p > 0.05).

**Table 2:** Nucleotide sequence variation in *Alvinella pompejana*.

Gene	EPR locality	*n*	*Hd (SD)*	*π_N _(SD)*	*θ_W _(SD)*	*D*	F*
*mtCOI*							
	northern	108	0.911 (0.014)	0.009 (0.000)	0.014 (0.004)	-0.983^NS^	-2.164 ^NS^
	southern	210	0.618 (0.040)	0.002 (0.000)	0.015 (0.004)	-2.564***	-4.330**
*GlobX*							
	northern	14	0.890 (0.060)	0.003 (0.000)	0.0034 (0.0017)	-0.872^NS^	-1.504^NS^
	southern	73	0.860 (0.031)	0.0054 (0.001)	0.012 (0.004)	-1.721**^+^**	-4.107**
*SAHH*							
	northern	26	0.471 (0.119)	0.002 (0.001)	0.004 (0.002)	-1.989*	-2.7696*
	southern	80	0.604 (0.064)	0.0031 (0.001)	0.011 (0.003)	-2.202**	-4.484**
*PGM*							
	northern	18	0.921 (0.030)	0.0056 (0.001)	0.007 (0.003)	-0.693^NS^	-1.437 ^NS^
	southern	74	0.725 (0.055)	0.0042 (0.001)	0.009 (0.003)	-1.836*	-3.950**

*n*, number of sequences; *Hd*, Haplotype diversity, *π_n _*nucleotide diversity; *θ_w_*, Watterson's theta per site from the number of segregating sites, *D*, Tajima's *D *index; F*, the Fu and Li index; *SD*, standard deviation; ^NS^: *P *> 0.10, ^+^: *P *< 0.10, *: *P *< 0.05, **: *P *< 0.01, ***: *P *< 0.001.

### Testing an isolation model with migration between the two geographic groups of *A. pompejana*

**Figure 4:**
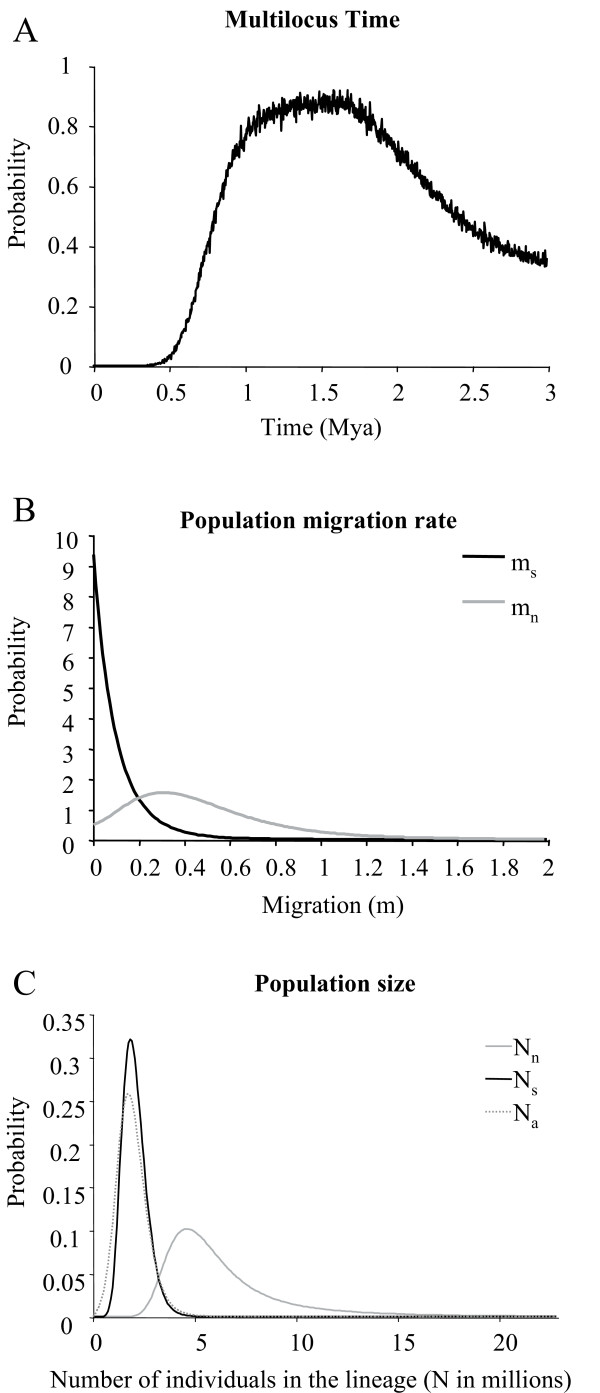
**Marginal posterior probability distribution for parameters estimated by the isolation-with-migration model**. Marginal posterior probabilities are estimated for (A) divergence time between the northern and southern EPR, (B) the forward-in-time migration rates between the northern and southern EPR, where m_s _represents south-to-north migration and m_n _north-to-south and (C) effective population sizes of the ancestral population (N_a_) and the two sister populations (i.e. N_n _and N_s _for the northern EPR population and the southern EPR population, respectively). Values estimated using IMa and 90% highest posterior density intervals of these parameters are presented in additional file 4.

The isolation-with-migration (IM) model of Hey and Nielsen [45] was used to test the occurrence of historical gene flow between northern and southern populations of *A. pompejana *or recent hybridisation and introgression following secondary contact. The estimated IM parameters are presented in additional file 4. Using all four genes, the split between the two groups of populations showed the highest likelihood values at divergence times of around 1-2 Mya (Figure 4A). IMa calculates migration rate based on coalescent analysis by looking from the present backward in time reversing the direction of migration when looking forward in time. The magnitude of migration from the north to the south (m_n_) and from the south to the north (m_s_) shows that gene flow occurred predominantly from the north to the south with no migration being detected in the opposite direction (Figure 4B).

Single-locus estimates of migration also exhibited asymmetry from the north to the south, although the parameters for *PGM*, *SAHH*, and *GlobX *were not significantly different from zero on the basis of their 90% highest posterior density (HPD) interval. This was clearly suggested by the allele networks for *PGM *and *GlobX *(Figure 3) for which a few alleles from northern clades were sampled in the southern EPR region. The mean time of migration events from the north to the south ranged from 25 000 to 300 000 years ago (Table 3), indicating that introgression occurred well after the initial isolation (~1.6 Mya) but not recently. The mean time of migration events (mtime) at *PGM *was, however, ten times lower than the two other loci suggesting more recent introgression (~23 000 years) at this locus.

**Table 3:** Number (m_#_) and mean time (mtime) of migration events, obtained from IM analyses.

Locus	m**_#s_**	m**_#n_**	mtime**_s _**(My)	mtime**_n _**(My)
*mtCOI*	0	0	-	-
*SAHH*	0	0-7	-	0.347
*GlobX*	0	0-7	-	0.388
*PGM*	0	1	-	0.023

Forward in time, labels (s) and (n) correspond to the south-to-north and north-to-south orientation of migration, respectively. For *SAHH *and *GlobX *genes, m**_# _**values correspond to the range of the posterior probability distribution without considering m = 0.

### Detection of hybrids at PGM between northern and southern populations

Based on the *PGM *network (Figure 3), we detected three individuals sampled on the southern EPR with at least one allele from a northern clade (two from 14°S and one from 7°25'S), but with the southern mitochondrial type. Owing to fixed substitutions and indels between the four *PGM *clades, all individuals collected at these sites were genotyped by combining RFLP and intron length polymorphisms. By comparing RFLP/indel genotypes and mitochondrial haplotypes of each individual, four 'hybrids' (33.3%) between the northern and southern groups were detected at 7°25'S, two (16.7%) at 14°S and only one (1.7%) at 13°N. No 'hybrids' were found at the other latitudes.

### Detecting demographic changes and/or selective effects

Tajima (*D*) and Fu and Li (F*) indexes were significantly negative for all loci in the south, but only *SAHH *displayed significant values in the north. All genes exhibited departures from neutral equilibrium between drift and mutation in the southern EPR, but not in the northern EPR. Similarly, for each nuclear gene, Fay and Wu tests were not significantly different from the neutral expectation in the northern populations, but were significantly negative in the southern populations.

**Figure 5:**
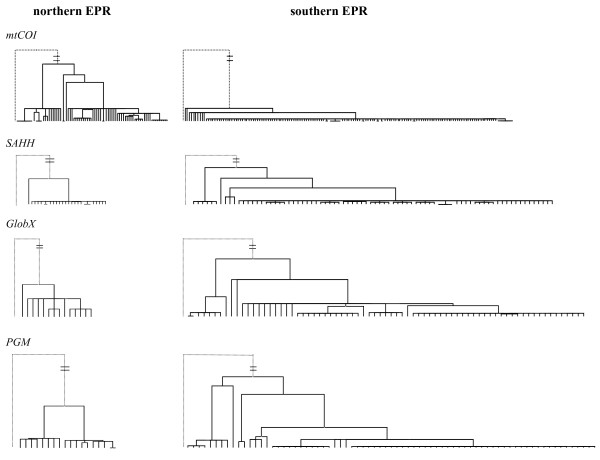
**Ultrametric neighbour-joining topologies of four genes in *A. pompejana *in the northern and southern EPR**. The outgroup *A. caudata *(dotted lines) was used to root the phylogenetic trees that were constructed from the most well-aligned parts of the four studied genes.

The *PGM *neighbour-joining tree displayed a topology similar to a gene experiencing balancing selection (i.e., the maintenance of 'old' lineages), whereas trees from the two other nuclear genes (*SAHH *and *GlobX*) had more star-like topologies typical of a selective sweep (Figure 5). The HKA multilocus test was significant using all genes (probability from χ^2 ^distribution < 0.00), indicating that at least one gene has experienced positive selection (or gene hitchhiking). After excluding *mtCOI*, the HKA test became nonsignificant (p χ^2 ^> 0.05), suggesting this gene could be experiencing strong purifying selection or a sweep. However, McDonald-Kreitman tests failed to reject neutral expectations at any locus (p > 0.05) when comparing *A. pompejana *polymorphism to the divergence of the *A. caudata *outgroup.

The Sweep_bott analyses performed on northern and southern groups of populations using the four loci are summarised in Table 4. When the analysis was performed over all genes, LRTs indicated that the bottleneck (M2) and selective sweep (M3) models both showed a significantly better fit with the dataset than the constant-size (M1) model. However, it was not possible to discriminate between the M2 and M3 hypotheses. When the analysis was performed on each gene individually, LRTs for M3 were all significantly better when compared with the M1 model for the southern populations, suggesting that a bottleneck might have occurred in this region. When the same analysis was done on the northern populations, LRTs for M3 were only significant for the *SAHH *gene, indicating that in addition to a bottleneck this gene may have undergone a selective sweep, at least in the northern populations. In effect, the M2 and M3 models are not mutually exclusive if a gene has experienced a moderate sweep in a bottlenecked population.

**Table 4:** MLEs and likelihood ratio tests computed using Sweep_bott software for bottleneck and selective models.

	Multilocus	Single-locus			
		***mtCOI***	***GlobX***	***SAHH***	***PGM***

northern EPR					
M1 MLE	-99.718	-37.728	-11.171	-14.341	-36.475
M2 MLE	-92.660				
M3 MLE	-90.460	-35.171	-9.712	-11.170	-34.407
LRT	M2-M1: 14.116				
	p < 0.005				
	M3-M1: 18.516	5.114	2.918	6.342	4.136
	p < 0.025	(NS)	(NS)	p < 0.025	(NS)
	M3-M2: 4.400				
	(NS)				

southern EPR					
M1 MLE	-159.367	-4.149	-57.112	-38.064	-60.042
M2 MLE	-153.211				
M3 MLE	-139.745	-0.290	-52.196	-30.557	-56.704
LRT	M2-M1: 12.312				
	p < 0.005				
	M3-M1: 39.243	7.718	9.830	15.014	6.677
	p < 0.005	p < 0.025	p < 0.01	p < 0.005	p < 0.005
	M3-M2: 23.282				
	p < 0.005				

M1, neutral model; M2, bottleneck model; M3, selective sweep model; NS, non-significant. Significances of the likelihood ratio tests (LRT) were tested according to a χ^2 ^table with a df equal to the difference of the number of parameters estimated for each model (df = 2, 8 and 6 for M2-M1, M3-M1 and M3-M2 multilocus LRTs, respectively, and df = 1 for the M3-M1 single-locus LRT).

## Discussion

### Emergence and strength of the equatorial barrier to gene flow

A genetic break at the *mtCOI *gene between the northern and southern EPR populations has been identified in previous studies by using a large number of hydrothermal vent species [28,29]. For some species, such as the gastropod *Lepetodrilus ovalis *and the bivalve *Bathymodiolus thermophilus*, a clinal distribution of the northern and southern *mtCOI *haplotypes was observed and attributed to the occurrence of a secondary contact zone between the northern and southern EPR [29]. In contrast, *A. pompejana *exhibited a clear equatorial break at this locus, raising the question of the permeability of this barrier to gene flow. The strength of the break between the northern and southern populations of *A. pompejana *appears to have significantly affected both allozyme and nuclear loci despite the recent formation of the barrier. Although the *SAHH *gene exhibited very little differentiation across this barrier, both the *GlobX *and *PGM *loci displayed a clear geographic separation of alleles. Divergence between northern and southern clades was estimated at ~1-2 Mya using the IMa multilocus estimation with very low migration rates. This estimate is in agreement with the shared vicariant event estimated at ~1.3 Mya from a series of vent species using approximate Bayesian computation on the *mtCOI *gene [29]. This isolation may be the consequence of the formation of multiple transform faults ~1-2 Mya in the region (Figure 1, [59-61]) reinforced by a strong transverse deep-sea current at the Equator [62].

**Figure 6:**
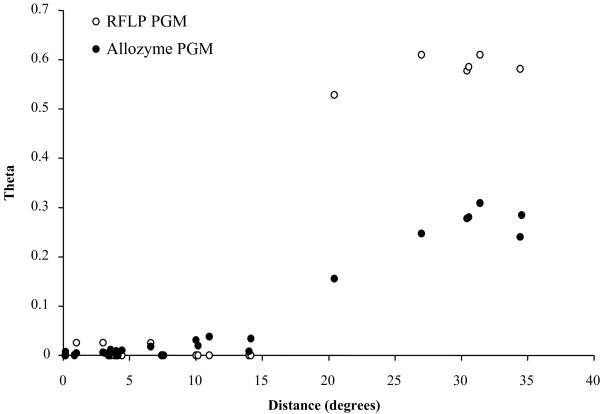
**Sensitivity of RFLP and allozyme PGM markers for detecting the equatorial genetic break**. The distance θ is plotted against the geographic distance between hydrothermal vent localities. The RFLP dataset shows a clearer departure from the isolation-by-distance model's expectation, indicating that markers to better detect the equatorial genetic break.

Slight, but significant differentiation at six allozyme loci across the equatorial barrier indicates that 1-2 My may not be sufficient to affect the net charge state of allozymes to create divergence, but likely reduced migration rates enough to modify allozyme frequencies by random genetic drift. This is particularly obvious in the distributions of allozymes and RFLP variants at the *PGM *locus (see Figure 6). Comparing patterns of genetic differentiation between PGM allozymes and DNA sequences indicated that allozymes are less sensitive at detecting the break. Moreover, the nearly complete replacement of the most frequent allele (100) in the northern populations by allele 78 in the south suggested that genetic drift is the main force acting to separate these populations at this locus. This equatorial break contrasts with the complete lack of genetic differentiation previously observed along thousands kilometres of vent fields on both sides of the EPR ([18], along the southern EPR in the present study) and confirms that isolation-by-distance by itself is not a valid argument to explain the observed decrease of gene flow for *A. pompejana*. In the absence of physical barriers, the species is able to disperse farther than expected based on moderate inter-field distances and the high bottom-current velocities [12]. This may be explained by either a developmental arrest of embryos in cold abyssal water masses and subsequent delay in metamorphosis [25], or the displacement of vent sites due to the movement of hydrothermal activity along the ridge axis, allowing bursts of colonisation by reconnecting previously isolated populations [21].

Questions addressing potential migration during an ongoing allopatric isolation process may be explored in two ways, depending on the primary cause for separation of populations (transform faults or bottom water circulation): (1) is there a progressive isolation of the north and south populations of *A. pompejana *by the offsetting of ridge segments at the Equator? or (2) is there emergence of a secondary contact between previously isolated populations due to the relaxation (i.e. attenuation of the transverse circulation) of the barrier?

### Permeability of the barrier

Variation in the magnitude of the genetic differentiation among loci has been widely reported during the process of allopatric speciation [63] and is commonly detected across barriers to gene flow [64-66]. This is mainly attributable to: (1) the sensitivity of the molecular method used to detect polymorphism, which in turn depends on the time scale at which the isolation is observed (i.e. the accumulation of non-synonymous mutations that change the net charge of a protein is a long process) [67], (2) the stochasticity of the coalescence process [68], and (3) the formation of a genetic barrier when secondary contacts occur [9,49,69]. Fitting an isolation-with-migration model [45] between the northern and southern EPR populations help to estimate both historical and contemporary migration and better understand the role of the equatorial barrier on the isolation process. Even if the estimated migration rates were not significantly different from zero, the shape of the posterior probability distribution of m_n _(north to south) was shifted in comparison to m_s _(see Figure 4B) and suggests that the barrier is porous. While migration was weak or absent at *mtCOI*, all nuclear genes displayed an asymmetric number of migration events across the Equator from north to south (Table 3), suggesting that allele migration can occur between the northern and southern groups. This was confirmed by detecting a few intermediate individuals at 7°25'S and 14°S between the northern and southern *PGM *groups.

The fact that gene flow is virtually absent for at least two loci (*mtCOI *and *PGM*) raises the question of whether the barrier is becoming increasingly impermeable to dispersal with time, or whether it represents a semi-permeable genetic barrier following recent secondary contact. In the first case, one can expect that most "migrating" alleles (i.e. alleles typifying one lineage found in the other one) would coalesce deeply in the evolutionary tree as they will represent incomplete lineage sorting. In the other case (emergence of a tension zone), all "migrating" alleles should coalesce shortly in the coalescence tree, leading to very low mtimes (times at which gene flow was maximum).

Mean times of migration at the three nuclear genes indicated that gene exchanges are probably not recent and may have been maximal ~20 000 to 300 000 years ago and support the hypothesis of incomplete lineage sorting and ancestral variation [see [49,70]], favouring an increasingly stronger barrier scenario. However, the high level of gene flow heterogeneity across both nuclear and mitochondrial loci favours the notion of a semi-permeable barrier to gene flow as previously defined by Harrison [71]. Natural selection is expected to prevent gene flow in regions of the genome linked to genetic incompatibilities and to generate interloci differences in migration rates [72]. To this extent, the restriction of 'hybrid' individuals as estimated from the *PGM *RFLP screening to a narrow zone located between 7°25'S-14°S could be indicative of the occurrence of a genetic barrier with differential selection against hybrids [69,73,74]. However, it may also simply reflect the fact that very few individuals are able to cross the geographic barrier by chance maintaining low frequencies according to the migration/drift equilibrium. Currently, the present number of nuclear loci is too small to strongly indicate the presence of a genetic barrier. Given that the vicariant event was quite recent (1-2 Mya), genetic incompatibilities and effective selection against hybrids may not have had time to arise supporting a model of progressive isolation of *A. pompejana *populations.

### Demographic expansion in the southern EPR hypothesis and evidence for a possible selective sweep at the *SAHH *gene

A comparative phylogeographic approach involving seven vent species using the *mtCOI *gene (including *A. pompejana*) revealed the occurrence of a concomitant demographic expansion in the southern EPR within the last 0.5 My [29]. In the present study, southern populations (but not necessarily northern populations) displayed significantly negative Tajima's D and Fu and Li statistics for all genes. Moreover, the bottleneck model (M2) was significantly better than the constant-size model (M1) in describing the genealogies associated with the southern populations. Assuming a similar distribution of nucleotide diversity across the studied genes (as expected for a demographic event such as expansion [54,56]), these results are in agreement with an expansion of southern populations, as hypothesised by Plouviez *et al. *[29]. Multiple extinction and recolonisation events possibly caused by a higher rate of tectonic rearrangements and eruptive phases in the southern EPR [75,76], would have favoured the recurrence of bottlenecks through time as previously seen from the long-term monitoring of targeted vent fields since 1991 [77,78]. This also fits well with the drastic change of allozyme frequencies at the PGM locus on each side of the barrier as genetic drift may be greatly enhanced by recurrent bottlenecks and/or founder effects [79,80]. However, IMa results provided a complete reverse scenario (Figure 4C) suggesting that only the northern populations increased in size since the population splitting. Although unexpected, this finding may be due to several causes: (1) the very high proportion of divergent allelic forms found in the northern populations, a situation typifying an 'old' diversified lineage, (2) the nearly equal sample sizes used in the analysis by suppressing both obvious recombinant alleles and randomly-selected alleles in the south, and (3) the possible non-neutral behaviour of at least one loci (i.e. the *SAHH*). The two former explanations seem unlikely. In the first case, the expectation would be an ancestral population size nearly similar to that of the northern one. In the second case, the Sweep_bott analysis (based on the same sequence dataset) would have validated the bottleneck scenario in the northern populations. The detection of a bottleneck in the southern populations does not preclude the possible additional effect of a selective sweep at any specific locus. The *SAHH *locus was the only locus that conformed to a selective sweep model on both sides of the EPR with virtually no genetic differentiation across the expected barrier, if an initial isolation step in allopatry is assumed. An advantageous allele (or a neutral allele linked to a gene under selection by hitch-hiking) is more likely to cross a genetic barrier and to spread within populations of the recipient lineage if the allele is advantageous for both lineages (e.g. [81,82]). Analyses of nucleotide polymorphism at this locus for northern and southern populations of *Alvinella caudata*, the most closely-related and syntopic species to *A. pompejana*, indicated a clear fixed divergence of alleles across the equatorial barrier analogous to divergences observed at both the *mtCOI *and the *PGM *loci in *A. pompejana *(S.P., unpublished data). This suggests that the lack of lineage sorting found in *A. pompejana *at the *SAHH *locus is probably not the result of a slow evolutionary rate at this locus but rather a case of high migration rate across the barrier. The spread of an allele across a permeable barrier is expected to be related to the magnitude of the selective advantage [83], suggesting that the sweep may be recent or very strong, even if the Fay and Wu test is only significantly negative in the southern EPR. Moreover, Faure *et al. *[49] proposed that diversifying selection helped to accelerate the evolution toward reciprocal monophyly at this specific gene in the Atlantic deep-sea vent mussels, suggesting that the *SAHH *gene may be indeed very sensitive to selection or linked to a potential positively selected gene.

## Conclusion

In summary, our multilocus study of *A. pompejana *populations confirmed the presence of an equatorial barrier, which plays an important role in structuring populations of the species *A. pompejana*, as proposed by Plouviez *et al. *[29]. This geographic barrier appears to be still permeable to migration. However, low migration rates that would occur primarily from north to south may also indicate that the barrier is becoming more and more impermeable suggesting that transform faults are a likely mean to promote allopatric speciation along oceanic ridges. In addition, discriminating a bottleneck model from a selective sweep model through the comparison of *mtCOI *and nuclear genes strongly supports the southern demographic expansion hypothesis previously suggested by a multispecies analysis using *mtCOI *only. Combining multilocus and multispecies analyses therefore allows us to propose that allopatry is probably one of the major forces promoting speciation and biodiversity within the vent fauna along the one-dimensional framework of oceanic ridges.

## Authors' contributions

SP carried out sequence acquisition and RFLP/indel genotyping as well as the molecular genetic analyses and drafted the manuscript. DLG genotyped individuals using allozymes. OL annotated the cDNA library of *A. pompejana*. FHL organised two of the three oceanographic cruises, helped sample specimens and provided helpful elements of discussion. DJ designed the study, carried out sampling, performed analyses with SP and edited the manuscript. All authors have read and approved the final manuscript.

## Authors' information

This study is part of SP's PhD thesis on the comparative phylogeography of deep-sea hydrothermal vent species along the East Pacific Rise (EPR). Her research involves multiple species and markers to examine speciation and demographic processes along the EPR. DLG is lab technician in charge of genotyping and biochemical analyses. OL works on comparative genomics and bioinformatics. FHL has broad knowledge of vent ecology and has spent most of his career working on the physiology of deep-sea hydrothermal vent species. DJ is a population geneticist working on the evolution and dispersal of deep-sea hydrothermal vent species. He has recently developed a genomic approach to better understand the role of gene adaptation in the evolution of vent species.

## Supplementary Material

Additional file 1 **Primers designed for DNA amplification on *Alvinella pompejana***. Table of sequences for forward and reverse primers used in the study. Click here for file

Additional file 2 **Size and exon-intron structure of the four sequenced genes**. Schematic drawings of the gene portion used in which exonic regions are represented by boxes. Click here for file

Additional file 3 **Allele frequencies, expected and observed heterozygosities and Fis estimated from enzyme loci across vent fields**. Table of allozyme frequencies and heterozygosities for each sampled population. Click here for file

Additional file 4 **Estimates and 90% Highest Posterior Density (HPD) intervals of demographic parameters from IMa multilocus analysis**. Table of 90% HPD intervals for population sizes, migration rates and time since population splitting estimated from the IMa Bayesian computations. Click here for file
