# Supplementary material for: Determining gene flow and the influence of selection across the equatorial barrier of the East Pacific Rise in the tube-dwelling polychaete Alvinella pompejana
Source: BMC Evol Biol. 2010 Jul 22;10:220. doi: 10.1186/1471-2148-10-220 (PMC2924869; doi:10.1186/1471-2148-10-220)
Supplement: Additional file 1 — Primers designed for DNA amplification on Alvinella pompejana. Table of sequences for forward and reverse primers used in the study. [file 1471-2148-10-220-S1.PDF]

Additional file 1

Primers designed for DNA amplification on *Alvinella pompejana*.

Gene	Primer sequences (5'-3')	T_a (°C)
mtCOI	**F:** TATTTGGTATTTGGGCAGGTC	57
	**R:** GATGGGTCGAAGAATGATGTG	
GlobX	**F:** ----AGTTGACTGAAGAACGCAGAGAAGCGGT	55
	**R:** ----CGCGAAGATCCTTGAAGTACTCTTGGTA	
SAHH	**F:** ----CTTGTCAACCTCGGCTGTG	57
	**R:** ----CTGCCTGCTCGTCTGTTAG	
PGM	**F:** ----CCCAGGTGGTCCAAATGC	57
	**R:** ----GTACAGATGTCAGCCTTCAGGTC	
Indel polymorphism	**F, F*:** GCCATGTTTTAATTGATTCTGTC	58
in *PGM* exon4	**R:** TCACTACAAAATGAGCTGATGTGC	

----: corresponds to the nucleotide '-bp tag' of primers for the Mark-Recapture sequence method. F, forward primer; F*, forward labelled primer IRD700™; R, reverse primer; T_a, annealing temperature
