# Supplementary figures and images for: Determining gene flow and the influence of selection across the equatorial barrier of the East Pacific Rise in the tube-dwelling polychaete Alvinella pompejana
Source: BMC Evol Biol. 2010 Jul 22;10:220. doi: 10.1186/1471-2148-10-220 (PMC2924869; doi:10.1186/1471-2148-10-220)

## Additional file 2

Size and exon-intron structure of the four sequenced genes.

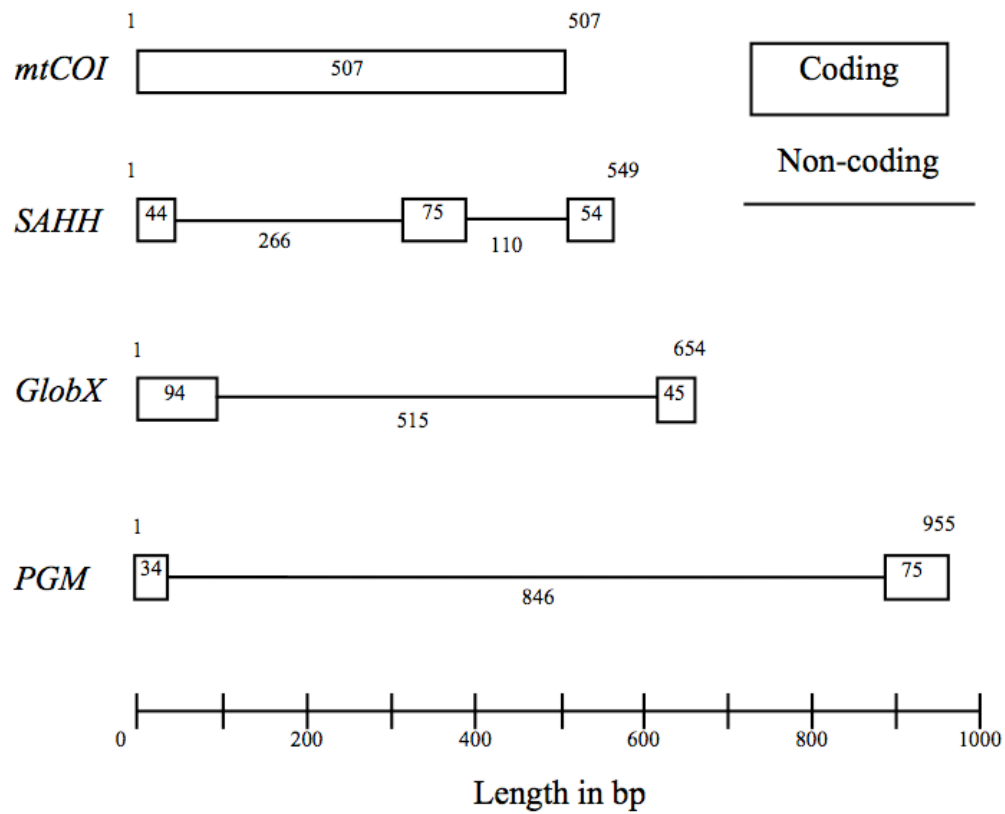

Supplement: Additional file 2 — Size and exon-intron structure of the four sequenced genes. Schematic drawings of the gene portion used in which exonic regions are represented by boxes. [file 1471-2148-10-220-S2.PDF]
