# Supplementary material for: Determining gene flow and the influence of selection across the equatorial barrier of the East Pacific Rise in the tube-dwelling polychaete Alvinella pompejana
Source: BMC Evol Biol. 2010 Jul 22;10:220. doi: 10.1186/1471-2148-10-220 (PMC2924869; doi:10.1186/1471-2148-10-220)
Supplement: Additional file 3 — Allele frequencies, expected and observed heterozygosities and Fis estimated from enzyme loci across vent fields. Table of allozyme frequencies and heterozygosities for each sampled population. [file 1471-2148-10-220-S3.PDF]

Additional file 3

Allele frequencies, expected and observed heterozygosities and Fis estimated from enzyme loci across vent fields.

**Locus**	**Vent field**						
	13°N	7°25'S	14°S	17°25'S	17°34'S	18°33'S	21°25'-33'S
**PGM**							
(N)	40	17	15	34	19	52	35
78	0.0349	0.3889	0.4000	0.4286	0.5000	0.4904	0.4286
90	0.3837	0.4167	0.5667	0.5571	0.4737	0.5096	0.5571
100	0.5116	0.1667	0.0333	0.0143	0.0263	0.0000	0.0143
112	0.0698	0.0278	0.0000	0.0000	0.0000	0.0000	0.0000
H exp.	0.5849	0.6466	0.5178	0.5057	0.5249	0.4998	0.5057
H obs.	0.5814	0.8889	0.8667	0.8857	1.0000	0.9808	0.8857
Fis	-0.128	0.022	0.104*	-0.030	-0.231	-0.281	0.139
**MPI**							
(N)	40	17	15	34	19	52	35
70	0.0125	0	0	0	0	0	0
92	0.0375	0	0	0	0.0263	0.0096	0
96	0.35	0.3235	0.1333	0.0882	0.1579	0.1058	0.0714
100	0.6	0.6765	0.8667	0.9118	0.8158	0.8846	0.9286
H exp.	0.5159	0.4377	0.2311	0.1609	0.3089	0.2062	0.1327
H obs.	0.675	0.2941	0.1333	0.1765	0.3684	0.1923	0.0857

Fis	-0.308	0.370*	0.451*	-0.097	-0.167	0.077	0.366

GPI

(N)	40	17	15	34	19	52	35
70	0.0125	0	0	0	0	0	0
100	0.9875	1	1	0.9118	0.9737	0.9904	0.9429
120	0	0	0	0.0882	0.0263	0.0096	0.0571
H exp.	0.0247	0	0	0.1609	0.0512	0.019	0.1078
H obs.	0.025	0	0	0.0588	0.0526	0.0192	0.0571
Fis	-0.012	-	-	0.644*	0.000	0.000	0.481

6PGD

(N)	40	17	15	34	19	52	35
80	0	0	0	0	0	0.0096	0
90	0.2	0.4118	0.2333	0.2206	0.3158	0.2019	0.1571
100	0.775	0.5588	0.7667	0.75	0.6842	0.6154	0.8286
110	0.025	0.0294	0	0.0294	0	0.1731	0.0143
H exp.	0.3588	0.5173	0.3578	0.388	0.4321	0.5505	0.2886
H obs.	0.3	0.4118	0.2	0.3235	0.5263	0.4808	0.2286
Fis	0.190	0.261	0.468	0.187	-0.192	0.136	0.222

IDH

(N)	40	17	15	34	19	52	35
100	0.9	0.8824	0.8333	0.9853	0.8947	0.9712	0.9429
120	0.0625	0.1176	0.1333	0	0.1053	0.0096	0.0571

140	0.0375	0	0.0333	0.0147	0	0.0192	0
H exp.	0.1847	0.2076	0.2867	0.029	0.1884	0.0564	0.1078
H obs.	0.15	0.2353	0.3333	0.0294	0.2105	0.0577	0.1143
Fis	0.204	-0.097	-0.129	0.000	-0.091	-0.013	-0.046

ACP

(N)	40	17	15	34	19	52	35
90	0.0125	0	0	0	0.0263	0.0096	0
100	0.9875	0.6765	0.9667	1	0.9737	0.9808	0.8143
120	0	0.3235	0.0333	0	0	0.0096	0.1857
H exp.	0.0247	0.4377	0.0644	0	0.0512	0.0379	0.3024
H obs.	0.025	0.5294	0.0667	0	0.0526	0.0385	0.3714
Fis	0.000	-0.150	0.000	-	0.000	-0.005	-0.214

LAP

(N)	40	17	15	34	19	52	35
90	0	0	0	0.0294	0	0	0
100	1	1	0.9333	0.9706	1	1	1
110	0	0	0.0667	0	0	0	0
H exp.	0	0	0.1244	0.0571	0	0	0
H obs.	0	0	0.1333	0.0588	0	0	0
Fis	-	-	-0.037	-0.015	-	-	-

TPI

(N)	40	17	15	34	19	52	35

82	0	0.0294	0	0.0294	0	0.0481	0.0143
90	0	0.0294	0	0.1471	0.0263	0.3269	0.0571
100	0.3875	0.8529	0.9333	0.6912	0.6842	0.6154	0.6857
110	0.5875	0.0882	0.0667	0.1324	0.2895	0.0096	0.2429
120	0.025	0	0	0	0	0	0
H exp.	0.5041	0.263	0.1244	0.4823	0.4474	0.512	0.4673
H obs.	0.6	0.1765	0	0.6176	0.4211	0.75	0.4
Fis	-0.232	0.358	1.000*	-0.257	0.086	-0.457	0.158
**HK**							
(N)	40	17	15	34	19	52	35
96	0.025	0	0	0.0441	0.0263	0.0096	0
100	0.5625	0.8824	0.6667	0.6618	0.7895	0.7308	0.7571
104	0.4125	0.1176	0.3333	0.2941	0.1842	0.2596	0.2429
H exp.	0.5128	0.2076	0.4444	0.4736	0.3421	0.3985	0.3678
H obs.	0.725	0.2353	0.2667	0.2059	0.3158	0.4231	0.4857
Fis	-0.388	-0.097	0.429	0.580***	0.104	-0.052	-0.308

N: number of individuals; H exp.: expected heterozygosity, H obs.: observed heterozygosity, *: $P < 0.05$, ***: $P < 0.001$.
