# Supplementary material for: Determining gene flow and the influence of selection across the equatorial barrier of the East Pacific Rise in the tube-dwelling polychaete Alvinella pompejana
Source: BMC Evol Biol. 2010 Jul 22;10:220. doi: 10.1186/1471-2148-10-220 (PMC2924869; doi:10.1186/1471-2148-10-220)
Supplement: Additional file 4 — Estimates and 90% Highest Posterior Density (HPD) intervals of demographic parameters from IMa multilocus analysis. Table of 90% HPD intervals for population sizes, migration rates and time since population splitting estimated from the IMa Bayesian computations. [file 1471-2148-10-220-S4.PDF]

Additional file 4

Estimates and 90% Highest Posterior Density (HPD) intervals of demographic parameters from IMa multilocus analysis.

Parameters	θ_n	θ_s	θ_a	t	m_s	m_n	N_n (x1000)	N_s (x1000)	N_a (x1000)	M_s	M_n	t (My)
Estimate (HiPt)	10.498	4.215	4.050	1.021	0.005	0.315	4085	1640	1576	0.026	0.038	1.589
Lower 90% HPD	5.703	2.397	1.571	0.477	0.005	0.005	2219	933	611	0.001	0.000	0.743
Higher 90% HPD	24.053	7.191	7.191	1.809	0.255	0.905	9360	2798	2798	19.385	3.727	2.816

For θ and N parameters, labels (n) and (s) correspond to the northern and southern groups of populations, respectively and label (a) corresponds to the parameters for the ancestral population. Forward in time, m_s and m_n correspond to south to north and north to south genes migration parameters, respectively.

$N_n = \theta_n /(4UG)$, $N_s = \theta_s /(4UG)$, $N_a = \theta_a /(4UG)$, $M_s = 2N1m_s$, $M_n = 2N2m_n$, t (years) = t/U, with U the geometric mean of the mutation rate per year across the loci and G the number of years per generation.
